# Common oncogenic mutations in colorectal cancer: drivers of carcinogenesis and potential therapeutic targets

**DOI:** 10.3389/fphar.2026.1807304

**Published:** 2026-04-30

**Authors:** Abaher O. ALTamimi, Noha M. Elemam, Shirin Hafezi, Maha Saber-Ayad, Iman M. Talaat

**Affiliations:** 1 Research Institute for Medical and Health Sciences, University of Sharjah, Sharjah, United Arab Emirates; 2 Department of Clinical Sciences, College of Medicine, University of Sharjah, Sharjah, United Arab Emirates; 3 College of Medicine, Cairo University, Giza, Egypt; 4 Pathology Department, Faculty of Medicine, Alexandria University, Alexandria, Egypt

**Keywords:** colorectal cancer, drug resistance, genetic alterations, microsatellite instability (MSI), mismatch repair (MMR), tumor microenvironment (TME)

## Abstract

Colorectal cancer (CRC) is marked by an intricate interaction of genetic mutations with the tumor microenvironment (TME). This review will provide updated insights into the effects of major mutations in CRC patients, including *MMR*, *APC, KRAS, BRAF, PIK3CA,* and *TP53,* on tumor progression and highlight their dynamic interactions with the TME, which can modulate, mask, or convert therapeutic sensitivity or resistance. Mutations in the *KRAS* and *BRAF* genes, for instance, have been associated with adverse outcomes and therapy resistance in CRC patients. Tumor profiling is significant for predicting prognosis and treatment responses, since mutation-specific crosstalk with the TME clarifies opportunities for personalized treatment strategies. Moreover, combination therapies targeting the multifaceted pathways of tumor cells and TME components have the potential to overcome drug resistance. New approaches in therapy are highly promising, especially in targeting the Wnt/β-catenin pathway, restoring APC function, and exploiting synthetic lethal interactions with truncated APC using next-generation small-molecule inhibitors, such as TASIN-1. More research is necessary to fully elucidate the interconnections among specific mutations, the TME, and treatment responsiveness to develop personalized therapies.

## 1 Introduction

**FIGURE 1:**
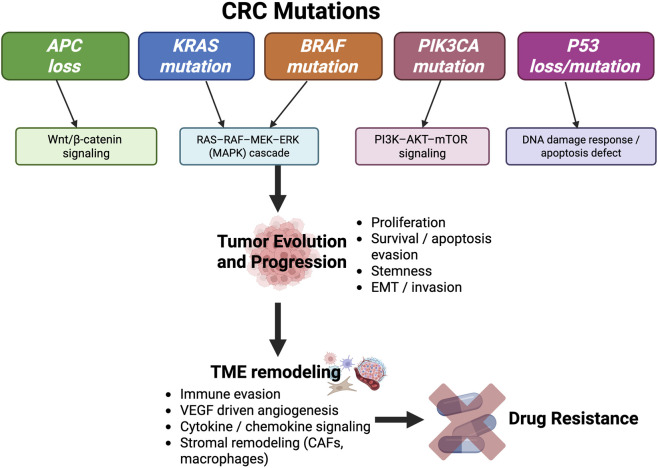
Key genetic mutations driving CRC progression and drug resistance. APC loss activates Wnt/β-catenin signaling; KRAS/BRAF mutations activate the RAS–RAF–MEK–ERK (MAPK) cascade; PIK3CA mutations activate PI3K–AKT–mTOR signaling; and TP53 loss impairs DNA-damage response and apoptosis. These pathways also promote immune evasion, angiogenesis, cytokine/chemokine signaling, and stromal remodeling, which contribute to clonal evolution and tumor progression, and ultimately lead to therapy resistance. APC: Adenomatous Polyposis Coli; KRAS: Kirsten Rat Sarcoma viral oncogene homolog; BRAF: B-Rapidly Accelerated Fibrosarcoma; PI3K: Phosphatidylinositol-4,5-bisphosphonate 3-kinase; P53: Tumor Protein p53.

Colorectal cancer (CRC) arises from complex interactions among epigenetic, environmental, and genetic factors, yielding multiple passenger and driver mutations (Fong et al., 2020). These genetic alterations significantly influence treatment outcomes and prognosis. Mutations have specific effects on disease behavior and treatment response (Loeb et al., 2019), potentially rendering chemotherapeutic drugs inactive (Figure 1). Several steps of CRC carcinogenesis are initiated or influenced by gene mutations, according to the Vogelstein model (Fearon and Vogelstein, 1990). In general, APC loss results in constitutive WNT/β-catenin signaling, which promotes adenoma formation and enhanced cancer cell proliferation (classic multistep model). On the other hand, KRAS-activating mutations activate downstream signaling cascades and induce cellular metabolic changes that ultimately promote tumor survival and growth (Sveen et al., 2018). Based on the COSMIC (Catalog of Somatic Mutations in Cancer) database, we selected the top-most frequently mutated genes in CRC. These include tumor suppressor genes (*APC, TP53*, and *SMAD4*) and oncogenes (*KRAS* and *BRAF*). Although not among the top mutated genes, we included the DNA repair genes (*MLH1, MSH2, and MSH6*), as they are well-recognized determinants of CRC prognosis and treatment (Loeb et al., 2019).

In addition to genetic mutations, epigenetic dysregulation contributes to the pathogenesis of CRC. DNA hypermethylation is one of the most frequent epigenetic changes (Zhao N. et al., 2024). In CRC, hypermethylation of CpG island promoters leads to the silencing of critical genes, including *MLH1* and other DNA repair genes, while global hypomethylation may result in chromosomal instability (Rico-Méndez et al., 2025). A subset of CRC exhibits promoter methylation, referred to as the CpG island methylator phenotype (CIMP), which is linked to MSI-high and hypermutated tumors (Ad et al., 2019).

CRC also has a high incidence of chromosomal instability and epigenetic changes, where the combination of specific mutational alterations in the tumor has both treatment and prognostic implications, including resistance to some therapies. An example is that resistance to targeted therapies against the epidermal growth factor receptor (EGFR), such as cetuximab and panitumumab, is commonly associated with *KRAS* mutations (Leite et al., 2025), whereas *BRAF V600E* mutations are implicated in resistance to conventional chemotherapy and consequently in poor prognosis (Fong et al., 2020; Caputo et al., 2019). On the other hand, mutations in the DNA mismatch repair genes can lead to microsatellite instability (MSI), which is associated with a better response to immunotherapies, such as immune checkpoint inhibitors (Loeb et al., 2019). Therefore, patient stratification in clinical trials based on mutational profiles enables a more precise evaluation of treatment efficacy in specific genetic subgroups. Thereby, understanding the CRC mutational landscape is critical for effective diagnostic, prognostic, and therapeutic approaches.

The treatment modalities for CRC include surgery, chemotherapy, radiation, immunotherapy, and targeted therapy (Chakrabarti et al., 2020). The common chemotherapy regimens are FOLFOX (5-fluorouracil, leucovorin, and oxaliplatin) and CAPOX (capecitabine and oxaliplatin) (Loree et al., 2018). Advanced or metastatic CRC (mCRC) may be treated with targeted therapies, such as bevacizumab, an anti-VEGF agent that inhibits angiogenesis, or cetuximab and panitumumab, which bind to the EGFR on tumor cells (Zhang et al., 2022). Immunotherapy with immune checkpoint inhibitors (ICIs), such as the PD-1 inhibitor pembrolizumab, is used for mismatch repair-deficient (dMMR/MSI-H) CRC cases (André et al., 2020). An interesting example is the effect of tumor microenvironment (TME) on modifying the response to immune checkpoint inhibitors in CRC with dMMR/MSI-H, where a dMMR/MSI-H tumor in a strongly TGF-β-rich stroma may still exclude T cells and resist immunotherapy (Tauriello et al., 2018).

Several reviews have comprehensively summarized the spectrum of oncogenic mutations in CRC, and others have focused on targeted therapeutic approaches. However, these topics are often addressed independently, with limited discussion on how specific genetic alterations actively remodel the TME and, in turn, dictate therapeutic resistance or sensitivity. This review uniquely integrates these perspectives by examining how major CRC mutations (*APC, KRAS, BRAF, PIK3CA,* and *TP53*) shape the immune, stromal, and extracellular components of the TME, and how this mutation-driven TME remodeling helps explain variability in responses to chemotherapy, targeted therapy, and immunotherapy. By linking mutation biology to TME dynamics and emerging mutation-guided combination strategies, this review provides a mechanistic framework that bridges molecular oncology, tumor immunology, and precision therapy in CRC.

## 2 Search strategy

The literature included in this review was identified through searches of PubMed, Scopus, and Web of Science using combinations of keywords related to colorectal cancer, oncogenic mutations (*APC*, *KRAS*, *BRAF*, *PIK3CA*, *TP53*, SMAD4), TME, immune regulation, and therapeutic resistance. Additional keywords related to mismatch repair deficiency and epigenetic alterations, including MLH1, MSH2, MSH6, DNA hypermethylation, MSI, and CpG island methylator phenotype (CIMP), were also incorporated to capture studies addressing molecular subtypes and epigenetic regulation in CRC. Priority was given to recent, high-quality, and mechanistically informative studies to provide a focused and up-to-date overview of the field. This targeted search strategy was designed to specifically capture studies that elucidate the mechanistic interplay between oncogenic mutations and tumor microenvironment remodeling, which represents the central focus and distinguishing feature of this review.

## 3 Tumor microenvironment

Over the past decade, a growing focus on TME has revealed that external factors significantly contribute to the development of chemoresistance (Ghosh et al., 2021). The complex tumor landscape, comprising both cellular elements (including cancer, immune, and stromal cells) and non-cellular components such as the extracellular matrix (ECM) and soluble factors, presents obstacles that foster chemoresistance. Cancer cells can alter the TME to enhance their survival (Ghosh et al., 2021). Different types of cells release exosomes, small lipid membranes with proteins and genetic material into the TME, which promote cancer cell invasion and survival (Wang et al., 2021). Furthermore, inflammatory factors within the TME facilitate epithelial-mesenchymal transition (EMT), metastasis, and resistance by expanding and recruiting cancer-associated fibroblasts (CAFs) and immune cells such as macrophages (Capaci et al., 2020a).

These mediators affect stromal cells in the TME, leading to the development of CAFs (Capaci et al., 2020a; Shakya et al., 2017). Among the diverse cell types present in the TME, CAFs are the most abundant and facilitate tumor progression through various pathways. They secrete factors such as angiopoietins 1 and 2, which promote angiogenesis, thereby supplying cancer cells with vital oxygen and nutrients (Fitzgerald and Weiner, 2020). In addition, CAFs engage with specific immune cells to uphold an immunosuppressive TME, thereby diminishing the effectiveness of ICIs (Hir et al., 2017; Liu T. et al., 2019; Piersma et al., 2020). CAFs play a crucial role in increasing ECM stiffness and impeding immune cell infiltration into the tumor stroma (Poltavets et al., 2018). These fibroblasts primarily originate from the activation of myofibroblasts, which are influenced by signals emitted by the tumor (Kaps and Schuppan, 2020). As a result, CAFs are characterized by elevated expression of α-smooth muscle actin (α-SMA), fibroblast activation protein, type I collagen, platelet-derived growth factor receptor-alpha/beta (PDGFRα/β), vimentin, and fibroblast-specific protein FSP-1 (S100A4). These molecules represent promising targets for drug delivery in anti-stromal therapies (Liu T. et al., 2019).

The investigation of CAFs and their role in secreting soluble factors is a prominent focus in TME research, particularly in understanding mechanisms of drug resistance. CAFs are recognized for their ability to release exosomes that contain Wnt ligands, which subsequently activate Wnt signaling pathways and contribute to drug resistance in differentiated CRC cells. Notably, suppression of Wnt secretion in Wnt3a-overexpressing CAFs has been shown to mitigate resistance (Hu Y. B. et al., 2019). Exosomes, which are small membrane-bound vesicles measuring between 30 and 100 nm in diameter, are secreted by a variety of cell types (Thakur et al., 2022). These vesicles serve as soluble mediators of intercellular communication, transferring active molecules such as microRNAs, mRNAs, and proteins between target cells (Hu J. L. et al., 2019). In CRC settings, CAF-derived exosomes play essential roles in cancer promotion, notably by promoting chemoresistance through the transfer of their molecular load to cancer cells (Thakur et al., 2022). Research has demonstrated that fibroblast-derived exosomes enhance chemoresistance in CRC by inducing CRC cells to transform into cancer stem cells (CSCs), which exhibit unique phenotypic and functional characteristics (Su et al., 2021). Notably, extracellular vesicles have been shown to play a critical role in regulating CSC properties, including self-renewal, cellular plasticity, and resistance to apoptosis. These vesicles facilitate intercellular communication by transferring bioactive molecules that reprogram recipient cancer cells toward a stem-like state, thereby promoting tumor progression and therapeutic resistance. Furthermore, CSC-associated extracellular vesicles help maintain a supportive tumor microenvironment, reinforcing survival signaling pathways and limiting the efficacy of conventional therapies (Su et al., 2021).

At the molecular level, Wnt proteins have been identified as the primary regulators of reprogramming in fibroblast exosomes (Luga et al., 2012; Koch et al., 2014; Chen et al., 2017). Exosomal Wnt proteins were found to increase Wnt activity and drug resistance in differentiated CRC cells. *In vitro* and *in vivo* experiments have shown that inhibiting Wnt release diminishes its effects (Hu Y. B. et al., 2019). Further research has shown that exosomal Wnt proteins from fibroblasts can promote dedifferentiation in cancer cells and enhance the chemoresistance of CRC. The authors suggested that interfering with exosomal Wnt signaling might enhance chemosensitivity and expand the therapeutic window (Hu Y. B. et al., 2019). Research has shown that CAFs in CRC cells release exosomes that increase miR-92a-3p levels, thereby stimulating activation of the Wnt/β-catenin pathway (Lin et al., 2024), preventing mitochondrial apoptosis while promoting CRC stemness, epithelial-mesenchymal transition (EMT), and resistance to 5-fluorouracil (5-FU) and oxaliplatin (Hu J. L. et al., 2019).

CAFs express long non-coding RNAs (lncRNAs) such as colorectal cancer-associated lncRNA (CCAL), which activates Wnt signaling to promote oxaliplatin resistance in CRC cells. Deng et al. (2020) demonstrated that CCAL promoted CRC chemoresistance to oxaliplatin therapy. CCAL is expressed in the stroma of the TME rather than within the tumor itself, and is transferred from CAFs to cancer cells via exosomes (Deng et al., 2020). Another mechanism of drug resistance is the expression of the drug efflux pump, p-glycoprotein (p-gp), encoded by the multidrug resistance (*MDR*) gene. CCAL-induced Wnt activation also increased MDR1 expression, potentially altering drug efflux (Tian et al., 2023). Further investigation revealed that MDR1/p-gp expression is upregulated through activation of Wnt/β-catenin signaling and suppression of the transcription factor activator protein-2α (AP-2α). Furthermore, epigenetic modifiers, such as histone H3 methylation and histone deacetylases, were found to contribute to the upregulation of CCAL in CRC (Huang et al., 2021).

Tumor-associated macrophages (TAMs) derived from the myeloid lineage primarily act to suppress immune responses. Within the TME, they release a variety of chemokines, including CCL17, CCL18, and CCL22, as well as growth factors such as vascular endothelial growth factor (VEGF) and tumor necrosis factor α (TNF-α) (Mamrot et al., 2019; Mi et al., 2021). These macrophages aid in the conversion of antitumor CD8^+^ T cells into regulatory T cells (Tregs), thereby promoting immune tolerance (Taniyama et al., 2019). Notably, TAMs have a dual function in CRC, either supporting or hindering tumor progression (Bockamp et al., 2020). Some studies have found that a high concentration of M1 macrophages (CD68^+^ TAMs) at the tumor’s invasive edge is associated with favorable outcomes, while other research linked the presence of M1 cells to advanced CRC stages, tumor invasion, and lymph node metastasis (Taniyama et al., 2019).

## 4 The role of mismatch-repair genes in colorectal cancer pathogenesis

Mismatch repair (MMR) genes are vital for rectifying errors during DNA replication, particularly in repetitive sequences or “microsatellites”. In CRC, the absence of crucial MMR genes-*MLH1, MSH2, MSH6*, and *PMS2*-leads to dMMR/MSI-H, which promotes the accumulation of mutations and tumor formation (Song et al., 2023). This repair system can be impaired by germline mutations, as seen in Lynch syndrome, or by epigenetic changes, such as hypermethylation of the *MLH1* promoter in sporadic CRC cases (Carnevali et al., 2023). dMMR/MSI-H results in a high mutation rate, particularly in genes that regulate cell growth and apoptosis, such as *TGFBR2* and *BAX*, thereby promoting tumor progression (Baretti and Le, 2018). These deficiencies may stem from inherited mutations, typical of Lynch syndrome, or sporadic epigenetic alterations, such as *MLH1* promoter hypermethylation, in sporadic CRC (Helderman et al., 2024).

**FIGURE 2:**
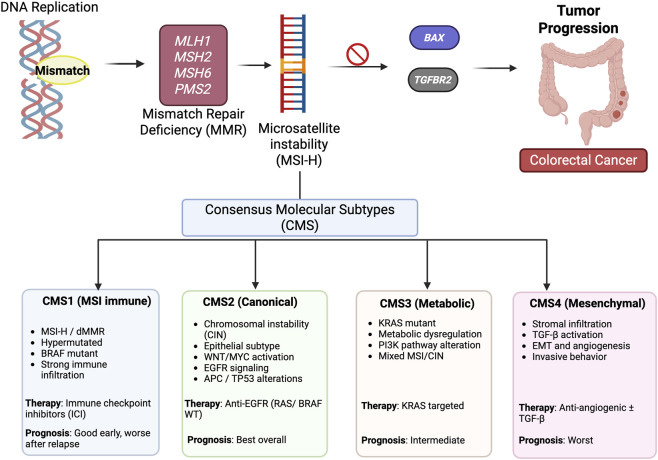
Molecular classification of CRC based on consensus molecular subtyping. CRC arises through the accumulation of genetic alterations, including MMR involving MLH1, MSH2, MSH6, and PMS2, leading to MSI and mutations in key target genes such as BAX and TGFBR2. CRC is divided into four consensus molecular subtypes (CMS). CMS1 (MSI–immune) is associated with dMMR/MSI-H, hypermutation, strong immune activation, frequent BRAF mutations, and treatment response to immune checkpoint inhibitors. CMS2 (canonical) is characterized by chromosomal instability, epithelial differentiation, and activation of the WNT/MYC and EGFR pathways and is associated with the best prognosis and response to anti-EGFR therapy in some patients. CMS3 (metabolic) is enriched for KRAS mutations and metabolic dysregulation, with intermediate prognosis and emerging targeted therapeutic strategies. CMS4 mesenchymal subtype, characterized by stromal infiltration, TGF-β activation, epithelial–mesenchymal transition, and angiogenesis, is predicted to be associated with the worst survival and could also benefit from anti-angiogenic and/or stromal-directed therapies. While CMS classification is not yet routinely applied in clinical decision-making, it provides important insights into CRC heterogeneity and therapeutic stratification. Bax: Bcl-2-associated X protein, MLH1: mutL homolog 1, MSH2: mutS homolog 2, MSH6: mutS homolog 6, PMS2: post-meiotic segregation increased 2, TGFBR2: transforming growth factor beta receptor 2.

CRC could be classified based on genetic and epigenetic signatures that impact outcomes and treatment (Valenzuela et al., 2021). The four consensus molecular subtypes (CMS) are: CMS1 (MSI-immune), with high MSI, intense immune activation, and frequent *BRAF* mutations; CMS2 (canonical), with chromosomal instability and Wnt and MYC pathway activation; CMS3 (metabolic), with metabolic dysregulation and frequent *KRAS* mutations; and CMS4 (mesenchymal), with stromal invasion, angiogenesis, and adverse survival (Thanki et al., 2017). Tumors with MMR deficiencies predominantly belong to the CMS1 subtype. It is well established that dMMR/MSI-H tumors are more sensitive to Immune checkpoint inhibitors (Willett et al., 2013). Awareness of the functions of *MMR* genes and of CRC molecular subtypes is critical for diagnosis, individualized therapy, and prediction of clinical behavior (Chowdhury et al., 2024). This awareness influences current CRC management, favoring precision medicine over a one-size-fits-all approach (Valenzuela et al., 2021). CRC classification based on CMS subtypes is summarized in Figure 2.

## 5 Adenomatous polyposis coli (APC) mutations in colorectal cancer

### 5.1 The mutation and its prevalence in colorectal cancer

The adenomatous polyposis coli *(APC)* tumor suppressor gene, when germline-affected, is a major genetic defect in familial adenomatous polyposis (FAP) syndrome, significantly increasing the risk of colon cancer. Somatic mutations in the *APC* gene are commonly seen in sporadic colon cancers. FAP is an autosomal-dominant inherited disorder caused by germline mutations in the *APC* gene. Germline mutations account for up to 80% of familial FAP cases (Tanabe et al., 2024). People with FAP often develop desmoid tumors (DTs) that originate from mesenchymal cells. The lack of *APC* disrupts Wnt signaling, promoting proliferation of mesenchymal stem cells (MSCs) and resulting in elevated β-catenin levels and sustained Wnt signaling (Yu et al., 2021). DTs also contain fibrocytes, which are involved in wound healing, angiogenesis, and fibrosis.

### 5.2 Molecular mechanism and impact on tumor microenvironment

APC regulates MSC growth and contributes to chemoresistance. MSCs can transform into CAFs, as previously reported (Sun et al., 2025). However, CAFs have multiple cellular origins (resident fibroblasts, pericytes, adipocytes, and EMT-derived cells) in addition to bone marrow MSCs (Huang et al., 2023; Rimal et al., 2022). CAFs are recognized for generating and depositing large quantities of dense ECM fibers, which disturb homeostasis. They also facilitate cancer cell invasion and spread by producing ECM proteins that promote metastasis, such as fibronectin and periostin, and by secreting matrix metalloproteases. This mechanism allows tumor cells to break away from the ECM and acquire mesenchymal traits (Telang, 2020).

Efforts to target the initial stages of the canonical Wnt/β-catenin pathway have demonstrated limited efficacy in treating cancers characterized by *APC* mutations (Chat et al., 2022). Cyclooxygenase-2 (COX-2), implicated in cell proliferation, differentiation, and tumor development, is identified as a downstream target of the Wnt pathway through β-catenin/TCF-mediated transcriptional activity. A reduction in APC levels has been associated with increased COX-2 expression in CRC cells (Kelson and Zaytseva, 2024). Similarly, the absence of the *COX-2* gene (*Ptgs2*) in CRC cells of Apc-mutant mice (Apc 716 (+/β) Ptgs2 (β/β)) resulted in a decrease in both the number and size of polyps (Fujishita et al., 2015). Consequently, inhibiting COX-2 was initially considered a potential adjunctive therapy for cancers with *APC* mutations. However, prolonged administration of sulindac (COX-1/COX-2 inhibitor) was found to induce tumor formation in the cecum and cause CRC recurrence due to drug-induced inflammation (Rodrigues et al., 2024). Research has shown that selective COX-2 inhibitors, such as MF-tricyclic and celecoxib, reduce polyp formation in Apc-mutant mice, which serve as models of human FAP (Rodrigues et al., 2024). Moreover, the effectiveness of celecoxib was further enhanced when combined with erlotinib, an EGFR inhibitor, resulting in a 96% decrease in polyps (Jin et al., 2019). However, owing to potential cardiovascular complications associated with long-term celecoxib use, COX-2 inhibitors are to be used in combination with erlotinib, yielding comparable outcomes in *Apc Min/+* mice (APC-driven models of colorectal tumorigenesis) (Liu et al., 2024). Similarly, sulindac has demonstrated the ability to diminish intestinal tumors in FAP patients, *Apc Min/+* mice, and other Apc-mutant models (Itano et al., 2009; Bohan et al., 2020).

### 5.3 Therapeutic targeting strategies

Initial approaches to inhibit Wnt/β-catenin signaling focused on restoring APC function via gene therapy. For example, studies have demonstrated that reintroducing APC into the intestines of Apc Min/+ mice reduced tumor burden (Dow et al., 2016; Arenas et al., 1996). Other studies have shown that introducing APC into CRC cells expressing only the truncated form of APC resulted in the translocation of β-catenin from the cytoplasm and nucleus to the plasma membrane, thereby suppressing cell growth and tumorigenicity. Moreover, restoration of functional APC increases cell-cell adhesion and yields a more adhesive epithelial morphology by modulating β-catenin localization and inhibiting Wnt/TCF transcriptional activity, thereby diverting β-catenin from the nucleus to the adherens junctions. (Wang T. et al., 2024). Notably, aminoglycosides and macrolides induce the synthesis of truncated proteins and restore APC function in cells with mutations. In CRC cells harboring a nonsense *APC* mutation, treatment with aminoglycosides reduced tumor growth, as these enhance drugs reading readthrough of nonsense codons (Ng et al., 2021). Furthermore, administration of aminoglycosides to Apc Min/+ mice resulted in reduced polyp size and extended lifespan. However, aminoglycosides and macrolides can be highly toxic and cause normal cells to produce toxic aggregates due to read-through of correctly placed stop codons. Although some aminoglycosides, such as tylosin, have been shown to be non-toxic, further studies on the mechanisms and toxicities of these compounds are needed (Graf et al., 2023). Nevertheless, restoration of APC through gene therapy or read-through mechanisms may be a successful therapeutic approach for *APC*-mutant cancers.

As previously indicated, the Wnt signaling pathway is implicated in CRC development, particularly through *APC* gene mutations. Given that the restoration of Apc can induce regression of CRC, researchers have explored the therapeutic potential of the Wnt pathway by developing small molecules that specifically target its components (Dow et al., 2016; Huang et al., 2009; Thorne et al., 2010). Although Wnt pathway inhibitors hold promise, their clinical use may be hindered by potential unintended toxic effects (Zhao X. et al., 2024). While APC plays roles beyond regulating the Wnt pathway, there is a notable lack of therapeutic approaches targeting the Wnt-independent functions of APC truncation.

Researchers have used a collection of isogenic human colonic epithelial cells (in both *in vitro* and *in vivo* models) to identify a small molecule, TASIN-1 (Truncated APC Selective Inhibitor-1). This compound selectively targets and inhibits the proliferation of cancer cells with APC truncations, while sparing cells with wild-type *APC* (Wang et al., 2019). Mechanistic investigations have shown that TASIN-1 exploits a synthetic lethal interaction between the cholesterol biosynthesis pathway and APC truncations, selectively inducing apoptosis in APC-mutant CRCs by depleting intracellular cholesterol and disrupting metabolic homeostasis (Wang T. et al., 2024). This phenomenon is partly attributed to the reduced activation of SREBP2 (sterol regulatory element-binding protein 2), a key regulator of cholesterol homeostasis, in APC-truncated cells exposed to cholesterol-depleting conditions induced by TASIN-1 treatment (Rasmi et al., 2025). Importantly, TASIN-1 induces selective apoptosis in APC-mutant CRCs through cholesterol biosynthesis inhibition without significant toxicity to normal colonic epithelium cells or APC-wild type cells. This selectivity highlights TASIN-1 and its derivatives as a targeted therapeutic strategy in CRC, harboring mutant APC (Theodoropoulos et al., 2020). Further studies are necessary to clarify the functional link between truncated APC and cholesterol regulation.

A tankyrase inhibitor can also be used to compensate for a mutated (truncated) nonfunctional APC. In canonical Wnt/β-catenin signaling, tankyrases (TNKS1/2) catalyze PARylation of the scaffold AXIN, promoting its ubiquitination and weakening the AXIN-APC-GSK3β destruction complex. Pharmacologic TNKS inhibition stabilizes AXIN, thereby reinforcing the destruction complex and driving β-catenin toward proteasomal degradation, which suppresses downstream TCF/LEF transcriptional output. Extending this mechanism, utilizing both *in vitro* and *in vivo* models, Zhu et al. describe a dual TNKS/USP25 inhibitor (UAT-B) that interferes with the TNKS-USP25 interaction, leading to a reduction in TNKS protein levels, further stabilizes AXIN1, and results in depletion of both cytoplasmic and nuclear β-catenin, overcoming multidrug resistance in TNKS-overexpressing CRC models (Zhu et al., 2024).

## 6 Kirsten Rat Sarcoma viral oncogene homolog (KRAS) mutations in colorectal cancer

### 6.1 The mutation and its prevalence in colorectal cancer

*KRAS*, a small GTPase of the RAS family located on chromosome 12, functions as a key regulator of cellular signal transduction pathways, controlling cell proliferation, differentiation, and survival (Shi et al., 2025). The most prevalent point mutations in *KRAS* occur at glycine 12 (G12), with subsequent mutations at glycine 13 (G13) and glutamine 61 (Q61) being less frequent (Meng et al., 2021). At the G12 position, there are 15 distinct point mutations, namely, G12A, G12D, G12F, G12K, G12N, G12S, G12V, G12Y, G12C, G12E, G12I, G12L, G12R, G12T, and G12W (Meng et al., 2021). KRAS mutations are present in approximately 35%–45% of CRC cases and occur frequently in both localized and metastatic disease (Takeda et al., 2025).

### 6.2 Molecular mechanism and impact on tumor microenvironment

In CRC, *KRAS* mutations lead to aberrant activation of signaling pathways, including the RAS/RAF/MEK/ERK pathway. Recent clinical and molecular evidence indicates that specific *KRAS* mutations are associated with activation of signaling pathways linked to liver metastasis by upregulating the MEK-Sp1-DNMT1-miR-137-YB-1-IGF-IR pathway, highlighting its role in metastatic CRC progression (Abdelga et al., 2025). Mechanistically, Chu et al. (2018) demonstrated that mutant *KRAS* transcriptionally activates *IGF-IR* expression through upregulation of Y-box-binding protein (YB-1) via the novel MEK-Sp1-DNMT1-miR-137 pathway in CRC cells (Chu et al., 2018). Notably, their research showed that KRAS-mediated colorectal liver metastasis can be suppressed in animal models by downregulating IGF-IR and YB-1, or by pharmacological inhibition of MEK. In human CRC, these genetic alterations have been linked to increased levels of certain interleukins and to the production of granulocyte-macrophage colony-stimulating factor (GM-CSF) (Pa et al., 2021). Notably, oncogenic *KRAS* has been shown to promote MDSC migration and reduce T cell infiltration into the CRC microenvironment by inhibiting interferon regulatory factor 2 (IRF2) expression, thereby activating the chemokine CXCL3 (Liao et al., 2019). This was demonstrated using a Kras/Apc/Trp53 mouse model and human CRC tissue (Liao et al., 2019). Furthermore, specific immune subpopulations, such as cytotoxic T cells and neutrophils, and the IFN-γ pathway were found to be suppressed in *KRAS*-mutant CRCs (Liao et al., 2019). Another study revealed that aberrant KRAS signaling was associated with differential expression of IL-23, IL-22, and IL-17 in the TME, thereby triggering immunological responses and inflammation-driven tumorigenesis (Hamarsheh et al., 2020). Although this has been demonstrated in previous studies, the precise mechanism linking interleukins and KRAS remains unknown.

### 6.3 Therapeutic targeting strategies

Despite the advantages of small-molecule cancer therapeutics, such as patient compliance, cost-effectiveness, and favorable pharmacokinetics, drug resistance remains a significant challenge to clinical efficacy, driven by mechanisms that cancer cells develop, including the emergence of mutations that reduce the long-term effectiveness of these drugs (Moore et al., 2020). For a long time, KRAS was labeled “undruggable” due to its small size, smooth surface, high affinity for its target, GTP, and highly flexible switch regions, which hindered the successful development of small-molecule inhibitors that selectively target it (Moore et al., 2020). To shift this paradigm, a recent approach, along with combination therapies, has been discovered to overcome such resistance. A possible approach is to develop covalent allosteric inhibitors that bind directly to the switch I/II pocket of mutant KRAS G12C at the A59 site, thereby disrupting protein-protein interactions (Chen et al., 2020). Another approach is developing GTP-competitive inhibitors that target the nucleotide-binding site. In June 2024, the U.S. FDA approved the combination of adagrasib plus cetuximab (Yaeger et al., 2024; U.S. Food & Drug Administration, 2020), and in January 2025, the combination of sotorasib plus panitumumab (Leite et al., 2025; U.S. Food & Drug Administration, 2021) for previously treated, KRAS G12C–mutated metastatic CRC. Other covalent KRAS G12C inhibitors, such as AMG510, MRTX849, JNJ-74699157, and GDC-6036, have advanced to clinical trials (Nagasaka et al., 2020; Liu P. et al., 2019) and show promise in circumventing the previously undruggable KRAS.

## 7 B-Rapidly Accelerated Fibrosarcoma (BRAF) mutations in colorectal cancer

### 7.1 The mutation and its prevalence in colorectal cancer

The *BRAF* gene is located on the long arm of chromosome 7 (7q34) and encodes a cytoplasmic serine/threonine protein kinase (B-Raf) that functions in the MAPK signaling pathway to regulate key cellular processes, including proliferation, differentiation, and cell survival (Perrone et al., 2024). *BRAF* mutations confer oncogenic properties and lead to persistent dimerization of the BRAF protein. This activates the RAF-MEK-ERK pathway, enhancing cell growth and suppressing apoptosis, thereby promoting cancer development (Śmiech et al., 2020). *BRAF V600E* mutations are found in approximately 12% of all cases of mCRC (Ciombor et al., 2022). Patients presenting with such a mutation tend to have a distinctive clinical profile and an unfavorable prognosis. The mutation is more commonly found in older female patients, with right-sided tumors, and is marked by poorly differentiated histology (Lièvre et al., 2020). *BRAF* mutations occur predominantly in sessile serrated adenomas and are associated with low-grade chromosomal instability, hypermethylation, and MSI. These occur in 40%–60% of MSI-positive metastatic CRC cases (Molina-Cerrillo et al., 2020).

### 7.2 Molecular mechanism and impact on tumor microenvironment

The complex interaction between cancer cells and their surrounding environment is crucial for tumor development and progression (Shen and Kang, 2018). This interaction is influenced by genetic alterations that modify the composition of the TME. A study showed that *BRAF* mutations, specifically the *BRAF V600E* mutation, significantly affected both the physical and chemical properties of the TME and were associated with a high concentration of FOXP3+ Treg cells and increased IL-1β production by CAFs (Śmiech et al., 2020). These characteristics are linked to poor outcomes due to the suppression of antitumor immune responses. Furthermore, *BRAF* mutations impair host defense by reducing the expression of immune and inflammatory response genes. The effectiveness of the immune system may be further diminished by the increased expression of immunosuppressive genes such as *HLA-G* (histocompatibility antigen, class I, G) and *PD-L1* (programmed death-ligand 1) (Kim et al., 2018). *BRAF* mutations play a crucial role in modulating the secretion of growth factors, cytokines, and chemokines, including VEGF (vascular endothelial growth factor), IL-1, IL-6, IL-10, CCL2, CCL4, CCL7, and CXCL8. This secretion modulates the recruitment of immune cells, including dendritic cells (DCs) and CAFs, which neutralize T-cell antitumor functions, thereby contributing to more aggressive tumor progression (Croce et al., 2019). This intricate mechanism promotes cancer cell proliferation, angiogenesis, and metastasis. Thereby, BRAF inhibitors can serve as a potential therapy by directly inhibiting cancer cell growth or modulating the immune microenvironment to suppress metastasis (Croce et al., 2019).

### 7.3 Therapeutic targeting strategies

Immunotherapy strategies targeting the tumor microenvironment in cases with *BRAF* mutations could offer personalized treatment options for CRC patients with tumors resistant to conventional therapies. The PETACC-3 randomized Phase III trial, which examined the effects of adding irinotecan to 5-fluorouracil/leucovorin in adjuvant CRC treatment, provided data to assess the prognostic significance of *BRAF* mutations (Caputo et al., 2019). Although recurrence-free survival differences between BRAF-mutant and wild-type CRC may be limited in certain cohorts, BRAF mutations, particularly in pMMR tumors, are strongly associated with significantly reduced overall survival and poorer clinical outcomes compared to wild-type disease (Dankner et al., 2025; Trunk et al., 2022). More recent real-world evidence from a single tertiary center reported a median overall survival of 18.2 months in patients with *BRAF* mutations, compared with 41.1 months in those with *BRAF* wild-type tumors (Kayhanian et al., 2018).

#### 7.3.1 Role of BRAF inhibitors

*BRAF* inhibitors (BRAFi), such as vemurafenib and dabrafenib, are targeted monotherapies that inhibit downstream BRAF signaling in the MAPK pathway (Proietti et al., 2020). This therapeutic approach has improved the treatment outcomes and overall survival of *BRAF-V600E* mutant melanoma (Robert et al., 2019). However, BRAFi monotherapy has been associated with poor efficacy in other cancers, such as mCRC. This discrepancy results from the intricate feedback loops and pathway crosstalk that mediate BRAFi resistance (Hanrahan et al., 2024). For instance, a compensatory activation of an upstream receptor tyrosine kinases (RTKs) through EGFR, reactivating the MAPK pathway regardless of BRAF inhibition (Corcoran et al., 2018). To overcome the intrinsic or acquired resistance to BRAFi monotherapies and to improve therapeutic outcomes, the current strategies have been shifted toward combinational therapies (BRAFi + MEKi) or BRAFi + (PI3K/AKT/mTOR or Wnt/β-catenin) inhibitors or by using anti-EGFR agents to either block the downstream signaling or the upstream feedback activation (Elez et al., 2025; Kopetz et al., 2019; Greger et al., 2012). Emerging therapeutic strategies, including the development of pan-RAF inhibitors capable of targeting both monomeric and dimeric RAF complexes, thereby overcoming the resistance driven by RAF dimerization and enhancing treatment efficacy in RAF mutant cancers (Manabe et al., 2026). Therefore, the complex resistance mechanisms observed in CRC remain an obstacle to treating mCRC with a *BRAF* mutation (Hanrahan et al., 2024; Elez et al., 2025; Kopetz et al., 2019).

## 8 Phosphatidylinositol-4,5-bisphosphonate 3-kinase (*PI3K*) mutations in CRC

### 8.1 The mutation and its prevalence in colorectal cancer

The phosphatidylinositol-4,5-bisphosphonate 3-kinase (PI3K) signaling pathway is a critical downstream signaling pathway involved in many cellular processes. It is activated in response to EGFR stimulation. Studies have shown that the PI3K signaling pathway plays a crucial role in both the progression and treatment response of various cancer types (He et al., 2021). PIK3CA is one of the most common somatic alterations in CRC and encodes the p110α catalytic subunit of class I PI3K. The most common hotspot mutations are located in exon 9 (E542K, E545K) and exon 20 (H1047R). These activating mutations lead to constitutive activation of the PI3K/AKT/mTOR signaling pathway, promoting tumor cell proliferation, survival, invasion, metastasis, and drug resistance (Wang H. et al., 2024). PIK3CA mutations have been found in 10%–30% of colon cancers, most commonly in conjunction with other molecular alterations, such as *KRAS* mutations (He et al., 2021). From a location perspective, *PIK3CA* mutations show a proximal predominance in CRC, with decreasing frequency toward the distal colon, and are associated with distinct clinicopathological features, including mucinous histology (Wang H. et al., 2024). Tumor suppressor protein phosphatase and tensin homolog deleted on chromosome 10 (*PTEN*) serves as a direct antagonist of the PI3K/AKT/mTOR pathway, and its mutational status can be associated with poor prognosis in CRC (Leiphrakpam and Are, 2024).

### 8.2 Molecular mechanism and impact on tumor microenvironment

Research suggested that the PI3K-AKT pathway is activated in the inflammatory TME of CRC (He et al., 2021). This activation can potentially result in the recruitment of different inflammatory cells, such as CD8^+^ T lymphocytes, by activating the NF-kB signaling pathway and producing prostaglandin E2 (He et al., 2021). This process could explain how these tumors develop mechanisms to evade T cell inflammation by expressing immune checkpoint markers (Essakly et al., 2020). Notably, *PIK3CA* mutations in CRC have been associated with phosphorylated AKT expression and with *KRAS* mutations (Voutsadakis, 2021). Furthermore, *PIK3CA* mutations have also been reported to be associated with other molecular features, such as MSI, CpG island methylator phenotype (CIMP), and *BRAF* mutations (Wang H. et al., 2024).

Studies have shown that *PIK3CA* mutations enhance both the PI3Kα (p110α) and AKT signaling pathways, leading to reduced apoptosis and increased cancer cell invasion (Wang H. et al., 2024). This also provides a rationale for patients with CRC harboring these mutations to have a poorer clinical prognosis. *PIK3CA* mutations also influence the CRC patients’ response to treatment. Conversely, studies have indicated that CRC patients with these mutations, particularly those with metastatic disease, have worse clinical outcomes and are less responsive to anti-EGFR monoclonal antibody therapy (Li et al., 2020). Therefore, analyzing *PIK3CA* genes before initiating anti-EGFR therapy may be a valuable approach for CRC patients, potentially leading to the development of targeted precision therapies.

### 8.3 Therapeutic targeting strategies

Aspirin, classified as a nonsteroidal anti-inflammatory drug (NSAID), has been shown to reduce both the occurrence and death rates of CRC (Agustsson and Bjornsson, 2025). Its action is mediated by the inhibition of cyclooxygenases (COX), leading to reduced production of prostaglandin E2 (PGE2), which subsequently boosts PI3K signaling (Jin et al., 2023). Additionally, some research suggests that CRC cases with *PIK3CA* mutations may respond to the Src inhibitor saracatinib (Din et al., 2022). It is noteworthy that CRC patients who are resistant to chemotherapy often exhibit a higher frequency of *PIK3CA* mutations. The resistance arises when colon cancer cells acquire stem-like properties via activation of the PI3K/AKT signaling pathway (Talukdar et al., 2023). Moreover, targeting HER2 and inhibiting the PI3K and MEK pathways have been shown to induce the death of cancer stem cells and cause regression of tumor xenografts, including those with *KRAS* and *PIK3CA* mutations (Mangiapane et al., 2022). Furthermore, combination therapy with palbociclib, a CDK4/6 inhibitor, and Gedatolisib, a PI3K/mTOR dual inhibitor, has shown synergistic anti-proliferative effects in CRC with MAPK and *PIK3CA* mutations. This drug combination could potentially offer benefits to patients with treatment-resistant CRC in the future (Lee et al., 2019).

#### 8.3.1 Role of PIK3CA inhibitors in CRC

*The PIK3CA* pathway is frequently dysregulated in various malignancies, including CRC, often through *PIK3CA* mutations or loss of tumor suppressors such as *PTEN* (Glaviano et al., 2023). Consequently, PI3K inhibition aims to impede downstream signaling components such as AKT and mTOR, thereby reducing tumor growth and survival signals (Leiphrakpam and Are, 2024). Both *in vitro* and *in vivo* models have demonstrated that PI3K inhibitors (PI3KCAi) can induce tumor cell apoptosis and inhibit proliferation; however, pathway redundancies and compensatory mechanisms in cancer cells can confer resistance to such monotherapies (Aslam et al., 2024). As a result, to enhance the efficacy of PIK3CAi, combination strategies that pair PI3K inhibitors with other targeted agents, such as MEKi, or with immunotherapies, are increasingly being explored (Wang H. et al., 2024).

Furthermore, genomic profiling to detect *PIK3CA* mutations has played a key role in identifying patients who may benefit from PI3K-targeted therapies, enabling a personalized approach in oncology (van de Haar et al., 2024). The value of PI3K inhibitors as a therapeutic approach, therefore, is not limited to their anti-tumoral effects but also to their ability to reverse resistance mechanisms and enhance responses in combination regimens; this is a promising new avenue in the field of precision oncology (Zhang et al., 2024).

## 9 Tumor Protein p53 *(TP53)* mutations in colorectal cancer

### 9.1 The mutation and its prevalence in colorectal cancer

Up to 60% of CRC cases harbor somatic TP53 mutations, which are linked to unfavorable clinical outcomes (Robles et al., 2016). TP53, often referred to as “the guardian of the genome,” is essential for maintaining cell-cycle control and genomic integrity. It is among the most extensively studied tumor suppressor genes and is located on chromosome 17p13.1. The p53 protein encoded by TP53 is composed of 393 amino acids and contains four functional domains. Its central sequence-specific DNA-binding domain (amino acids 101–306) mediates DNA binding and is commonly disrupted in mutant forms of p53, thereby compromising its normal biological activity (Mi et al., 2021; Cho et al., 1994; Li et al., 2019).

### 9.2 Molecular mechanism and impact on tumor microenvironment

The p53 protein plays a vital role in both tumor initiation and TME remodeling by interacting with immune cells (Ghosh et al., 2021). *TP53* DNA alterations can transform p53 from a tumor-suppressive protein into a mutant protein that promotes malignant evolution of CRC. In addition, some missense TP53 mutants acquire gain-of-function activities that promote aggressive tumor behavior and remodel the TME. Mutant p53 has been shown to suppress the cGAS-STING-TBK1-IRF3 innate immune pathway and attenuate antitumor immune surveillance (Ghosh et al., 2021). In CRC, mutant p53-derived exosomes carrying miR-1246 can reprogram macrophages toward a tumor-supportive phenotype (Cooks et al., 2018). These findings suggest a direct link between TP53 mutations, aberrant p53 protein function, immune and TME remodeling in CRC.

### 9.3 Therapeutic targeting strategies

Studies have demonstrated that using HDM201, an inhibitor of the E3 ubiquitin ligase MDM2, to block p53 degradation increases CD8^+^ T cells and the CD8/Tregs ratio, thereby enhancing immune-mediated antitumor responses (Wang et al., 2021). MDM2 is an E3 ligase that binds with and ubiquitinates p53. Small-molecule MDM2–p53 antagonists prevent this interaction and restore p53-mediated cell-cycle arrest and apoptosis, but only in the presence of wild-type TP53 (Siriki et al., 2025). Combining HDM201 with PD-L1 blockers had increased the number of complete tumor regressions in mouse tumor xenograft models. Thus, restoring p53 or preventing its degradation could be a promising strategy for cancer treatment. Furthermore, p53 mutations affect the composition of the tumor secretome, which comprises ECM components, remodeling enzymes, exosomes, and soluble mediators, such as growth factors, cytokines, and chemokines, found in the TME (Qiu et al., 2024).

Research has shown that inhibition of p53 in stromal cells, including CAFs, can promote immune escape and carcinogenesis (Blagih et al., 2020). Furthermore, hypoxic conditions within the TME induce the expression of oncogenic microRNAs that suppress TP53 signaling, thereby promoting tumor progression (Capaci et al., 2020b). It is worth noting that miRNA-30d primarily affects TME cells in this context, as its impact on mutated p53 is limited owing to its relatively high stability compared to the non-mutant form (Blagih et al., 2020). Studies have shown that p53 regulates innate immune cells, including TAMs. In CRC, the presence of p53 in cancer cells is associated with M2 macrophages (CD204+ TAMs) and tumor blood vessel density, though additional research is needed to clarify the underlying mechanisms (Taniyama et al., 2019). As a result, p53 plays a role in immune surveillance within the TME in CRC, and its dysfunction may promote tumor progression.

A list of major CRC mutations, their biological effects, associated pathways, and targeted therapies currently under investigation or in clinical use is shown in Table 1.

**TABLE 1:** List of major CRC mutations, types, their biological effects, associated key pathways, and targeted therapies.

Gene	Mutation type	Biological Effects	Key Pathways	Targeted Therapies (Approved/Investigational)	Key References
APC	Loss of Function	Stabilization of β-catenin → uncontrolled proliferation and tumor initiation	Wnt/β-catenin	-Tankyrase inhibitors (preclinical) -Wnt inhibitors -COX-2 inhibitors	Li et al. (2024)
KRAS	Activating (G12, G13, Q61)	Constitutive RAS activation → continuous proliferation and survival signaling	MAPK (RAS–RAF–MEK–ERK)	-KRAS G12C inhibitors (adagrasib, sotorasib) + EGFR inhibitors -MEK inhibitors (trials)	Takeda et al. (2025)
BRAF	V600E	Constitutive kinase activation → MAPK hyperactivation, aggressive phenotype	MAPK pathway	BRAF inhibitors (vemurafenib, dabrafenib) + EGFR inhibitors; BRAF + MEK combos; pan-RAF inhibitors	Caputo et al. (2019)
PIK3CA	Activating (exon 9/20)	AKT activation → survival, metabolism, therapy resistance	PI3K–AKT–mTOR	PI3K inhibitors (alpelisib); PI3K + MEK combos; aspirin (biomarker-selected benefit)	Wang et al. (2024b)
TP53	Loss or gain-of-function	Impaired apoptosis, genomic instability, tumor progression	p53/cell cycle/DNA damage response	MDM2 inhibitors (e.g., HDM201); p53 reactivation (experimental); combination immunotherapy	Mi et al. (2021)
SMAD4	Loss-of-function	Disrupted TGF-β signaling → metastasis and invasion	TGF-β pathway	TGF-β inhibitors (experimental)	Fang et al. (2021)
PTEN	Loss-of-function	Increased PI3K signaling and resistance to targeted therapies	PI3K–AKT–mTOR	PI3K/mTOR inhibitors; combination targeted therapy	Salvatore et al. (2019)

## 10 Emerging technologies and clinical implications of CRC mutations

Recent advances in genomic technologies have further enhanced our understanding of TME interactions in CRC. Single-cell sequencing enables high-resolution profiling of tumor, immune, and stromal cell populations, uncovering mutation-associated transcriptional programs that may be obscured in bulk analyses (Yu et al., 2025). Complementary to this, spatial transcriptomics preserves tissue architecture and enables *in situ* mapping of cellular interactions, facilitating the identification of spatial niches associated with immune exclusion and therapeutic resistance (Liu et al., 2026). Moreover, integrative multi-omics approaches that combine genomic, transcriptomic, epigenomic, and proteomic data are increasingly used to bridge the gap between genotype and phenotype, providing deeper insights into how specific mutations drive TME remodeling and influence treatment response (Sibilio et al., 2025).

**FIGURE 3:**
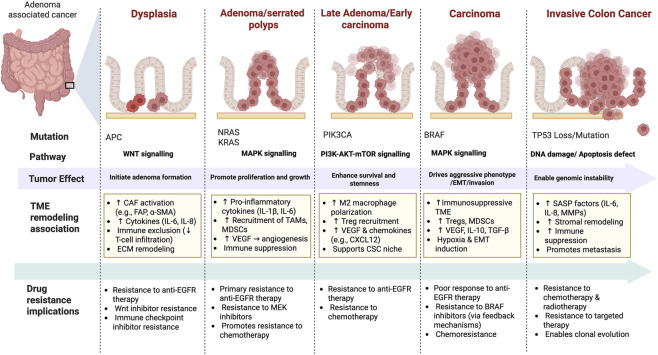
Integrative scheme of CRC mutations, TME remodeling, and drug resistance mechanisms. CRC progression results from a stepwise accumulation of driver mutations. These events are accompanied by TME remodeling, including recruitment and activation of cancer-associated fibroblasts, cytokine/chemokine signaling, immunosuppression, angiogenesis, and extracellular matrix reorganization. At the same time, they promote therapy resistance, including resistance to targeted therapies and chemo/radiotherapy. CAF, cancer-associated fibroblast; TAM, tumor-associated macrophage; MDSC, myeloid-derived suppressor cell; Treg, regulatory T cell; cancer stem cell; ECM, extracellular matrix; VEGF, vascular endothelial growth factor; SASP, senescence-associated secretory phenotype; EMT, epithelial–mesenchymal transition; TME, tumor microenvironment; MAPK, mitogen-activated protein kinase; PI3K, phosphoinositide 3-kinase; AKT, protein kinase B; mTOR, mechanistic target of rapamycin; EGFR, epidermal growth factor receptor.

Mutated genes can lead to the development of therapeutic resistance in CRC. As previously discussed under the sections for each gene, those mutations can perpetuate resistance through their effects on the TME (Oh et al., 2025; Favier et al., 2025; Han et al., 2026). APC mutations favor immune exclusion and impaired T-cell recruitment through a WNT/β-catenin dominant background. This creates cold TME that may limit immunotherapy responsiveness and enhance therapy resistance (Janeeh et al., 2025). KRAS mutations are strongly associated with resistance to anti-EGFR therapy due to constitutive downstream MAPK signaling, regardless of receptor blockade. Beyond this intrinsic effect, KRAS mutant tumors also remodel the TME through myeloid suppression and disrupted macrophage-T-cell crosstalk, reinforcing immune escape and reducing treatment efficacy (Leite et al., 2025; Zhang et al., 2025). BRAF V600E mutant CRC is initially targetable, but resistance often develops through MAPK pathway reactivation. BRAF mutation often accompanied by T-cell dysfunction rather than effective immune control (Guerrero et al., 2023). PIK3CA mutations encourage persistent PI3K/AKT/mTOR signaling, supporting survival, invasion, and drug resistance. This pathway can serve as a bypass track when other pathways are blocked; additionally, it influences the TME through paracrine signaling and inflammatory reprogramming (Wang H. et al., 2024). TP53 mutant CRC contributes to the resistance through impaired apoptosis and enhanced genomic instability. P53 dysfunction also alters angiogenic and immune signaling, resulting in tumor survival (Yan et al., 2025; Ruzzo et al., 2025). A summary of how the specific mutations mechanistically remodel the TME and influence immune evasion or drug resistance is shown in Figure 3.

From a clinical perspective, CRC molecular profiling plays a major role in biomarker-based stratification and therapy selection. Current practice for mCRC emphasizes assessing KRAS, BRAF, HER2, and dMMR/MSI-H status, as these biomarkers have therapeutic implications. RAS mutant CRCs do not respond to anti-EGFR monoclonal antibodies, while BRAF V600E-mutant tumors respond better to the combination therapy. Conversely, dMMR/MSI-H tumors respond to immune checkpoint inhibitors, and HER2-amplified tumors may benefit from anti-HER2 approaches when RAS and BRAF are wild type (Benson et al., 2024; Whitmer et al., 2026; Cervantes et al., 2023). Thereby, the molecular classification is not only prognostic, but also predictive of the treatment path.

Based on this molecular stratification, recent therapeutic progress has been focused on targeting specific oncogenic drivers, particularly KRAS G12C, through combination strategies designed to overcome intrinsic resistance to monotherapy. Adagrasib plus cetuximab was evaluated in the phase I/II KRYSTAL-1 study in patients with previously treated unresectable or metastatic KRAS G12C-mutated CRC after fluoropyrimidine-, oxaliplatin-, and irinotecan-based chemotherapy (and a VEGF inhibitor when eligible). Among the 94 treated patients, the combination achieved an objective response rate (ORR) of 34%, a median duration of response of 5.8 months, a median progression-free survival (PFS) of 6.9 months, and a median overall survival (OS) of 15.9 months. This regimen received FDA accelerated approval on 21 June 2024, and the confirmatory phase III KRYSTAL-10 study is ongoing in the second-line setting (Yaeger et al., 2024; Food and Administration, 2024; Tabernero et al., 2021). For sotorasib-based therapy, sotorasib plus panitumumab first demonstrated activity in the phase Ib CodeBreaK 101 study in chemotherapy-refractory KRAS G12C-mutated metastatic CRC, in which 48 patients were treated; the dose-expansion cohort showed a confirmed ORR of 30%, median PFS of 5.7 months, and median OS of 15.2 months. Further results from the randomized phase III CodeBreaK 300 trial in 160 previously treated patients, in which sotorasib 960 mg plus panitumumab improved PFS (5.6 vs. 2.0 months; HR 0.48) and increased ORR (26% vs. 0%) compared with the investigator’s choice treatment. In the final OS analysis, median OS was not reached with sotorasib 960 mg plus panitumumab, compared with 10.3 months with standard therapy, although the difference was not statistically significant. This combination received FDA approval on 16th January 2025 (Kub et al., 2024; Food and Administration, 2025; Pietrantonio et al., 2025).

## 11 Conclusion

CRC is not solely a disease driven by isolated genetic mutations but rather a dynamic malignancy shaped by continuous and reciprocal interactions between oncogenic alterations and the TME. Throughout CRC progression, key driver mutations, including *APC, KRAS, BRAF, PIK3CA, TP53*, and defects in mismatch repair, do more than promote uncontrolled proliferation; they actively remodel the immune, stromal, and extracellular architecture of the tumor niche. This mutation-driven TME remodeling largely explains the heterogeneity in therapeutic responses, the development of drug resistance, and the variability in clinical outcomes observed among CRC patients.

Loss of *APC* initiates Wnt/β-catenin–dominant signaling that favors immune exclusion and stromal activation. *KRAS* and *BRAF* mutations not only sustain MAPK pathway activation but also promote immunosuppressive cytokine networks, myeloid cell recruitment, and T-cell dysfunction. *PIK3CA* mutations confer survival advantages via PI3K/AKT/mTOR signaling while contributing to inflammatory and immunomodulatory changes within the TME. Meanwhile, *TP53* mutations alter innate immune signaling, exosomal communication, and macrophage polarization, further reinforcing a tumor-supportive microenvironment. In parallel, mismatch repair deficiency and MSI status influence immune infiltration and responsiveness to immune checkpoint inhibition. Together, these insights highlight that therapeutic resistance in CRC often arises not only from intrinsic tumor cell signaling but also from mutation-guided reprogramming of the TME.

This integrated understanding carries important clinical implications. Molecular profiling in CRC is no longer only prognostic but fundamentally predictive, guiding treatment decisions such as the use of anti-EGFR therapy, immune checkpoint inhibitors, BRAF-targeted combinations, and emerging KRAS G12C-directed regimens. Moreover, advances in single-cell technologies, spatial transcriptomics, and multi-omics integration are uncovering spatial and cellular contexts in which these mutations operate, offering unprecedented opportunities to design mutation-informed, TME-directed combination therapies.

Emerging strategies that target Wnt signaling in APC-mutant tumors, exploit synthetic lethality (e.g., TASIN-1), inhibit KRAS G12C in combination with EGFR blockade, overcome BRAF inhibitor resistance through rational combinations, and integrate PI3K or p53-directed approaches with immunotherapy exemplify the future of precision oncology in CRC. These approaches move beyond single-pathway inhibition toward coordinated targeting of both tumor-intrinsic mutations and the microenvironmental consequences of these mutations. Ultimately, effective CRC management will require therapeutic strategies that acknowledge this duality: cancer as a genetic disease and as a microenvironment-driven ecosystem. By bridging molecular oncology, tumor immunology, and stromal biology, this review underscores a paradigm in which understanding mutation-TME crosstalk is essential to overcoming resistance and improving patient outcomes. Future research focused on translating this mechanistic knowledge into clinically actionable combination therapies holds the promise of truly personalized treatment for patients with CRC.
